# Alternative phenotypes of male mating behaviour in the two-spotted spider mite

**DOI:** 10.1007/s10493-013-9673-y

**Published:** 2013-02-20

**Authors:** Yukie Sato, Maurice W. Sabelis, Martijn Egas, Farid Faraji

**Affiliations:** 1 Institute for Biodiversity and Ecosystem Dynamics, University of Amsterdam, P.O. Box 94240, 1090 GE Amsterdam, The Netherlands; 2 National Institute for Agro-Environmental Sciences, 3-1-3 Kannondai, Tsukuba, Ibaraki, 305-8604 Japan; 3 Japan Society for the Promotion of Science, Tokyo, Japan; 4 Mitox Consultants, Science Park 406, 1098 XH Amsterdam, The Netherlands

**Keywords:** Alternative tactics, Male antagonism, Male competition, Intrasexual selection, *Tetranychus urticae*

## Abstract

Severe intraspecific competition for mates selects for aggressive individuals but may also lead to the evolution of alternative phenotypes that do not act aggressively, yet manage to acquire matings. The two-spotted spider mite, *Tetranychus urticae*, shows male mate-guarding behaviour and male–male combat for available females. This may provide opportunity for weaker males to avoid fighting by adopting alternative mating behaviour such as sneaker or satellite tactics as observed in other animals. We investigated male precopulatory behaviour in the two-spotted spider mite by means of video-techniques and found three types of male mating behaviour: territorial, sneaker and opportunistic. Territorial and sneaker males associate with female teleiochrysales and spend much time guarding them. Territorial males are easily disturbed by rival males and engage themselves in fights with them. However, sneaker males are not at all disturbed by rival males, never engage in fights and, strikingly, never face attack by territorial males. Opportunistic males wander around in search of females that are in the teleiochrysalis stage but very close to or at emergence. To quickly classify any given mate-guarding male as territorial or sneaker we developed a method based on the instantaneous response of males to disturbance by a live male mounted on top of a brush. We tested this method against the response of the same males to natural disturbance by two or three other males. Because this method proved to be successful, we used it to collect territorial and sneaker males, and subjected them to morphological analysis to assess whether the various behavioural phenotypes are associated with different morphological characters. However, we found no statistical differences between territorial and sneaker males, concerning the length of the first legs, the stylets, the pedipalps or the body.

**Electronic supplementary material:**

The online version of this article (doi:10.1007/s10493-013-9673-y) contains supplementary material, which is available to authorized users.

## Introduction

Males often engage in fights with conspecific males to secure mating with available females and ultimately to ensure fathering offspring (Andersson 1994). Intrasexual selection generally favours males competitive enough to win the fight. However, less competitive males frequently get access to females by performing alternative male mating behaviour such as sneaking, female mimicking and taking a satellite position (e.g. Gross 1996; Brockmann 2001; Radwan 2009; Taborsky et al. 2008). Sneaking behaviour by smaller, less competitive males is well known in the family Salmoninae (Esteve 2005). If the smaller males fight with larger males head-on, they lose the fight and cannot father offspring at all (Esteve 2005). The smaller males instead take a strategic position away from large, fighting males and wait for the moment of spawning. At the moment of egg release, the smaller males take a position closer to the eggs and release sperm when the smaller males are close enough and not attacked by larger males, usually because males are engaged in fights with other large males. In this way, the smaller males gain fertilization success without having to win a fight over a female.

The two-spotted spider mite, *Tetranychus urticae* Koch, is a polyphagous herbivore feeding on leaves of plants and it is a pest of various crops all over the world. Because of its importance as a pest, its ecology and behaviour have been well investigated (Helle and Sabelis 1985) including its mating behaviour (e.g. Cone 1985). Male precopulatory behaviour of the spider mite consists of mate guarding behaviour and male–male combat and is affected by several factors, such as sex pheromones, female condition and body size (Potter et al. 1976a, b; Royalty et al. 1994; Tien et al. 2011). However, the existence of alternative phenotypes for male mating behaviour has not been studied. We expect such behavioural alternatives in the two-spotted spider mite, because as observed by Potter et al. (1976a, b) male combat for available females is intense.

In this paper, we observed precopulatory behaviour in males of the two-spotted spider mite using video-techniques and investigated differences in premating behaviour among the males. In this way, we were able to describe three behavioural phenotypes. To enable future research on the conditions promoting their coexistence, we also developed and tested a relatively quick method for identifying these behavioural phenotypes. According to the literature (e.g. Emlen 1997; Radwan^1993^, ^2003^), alternative male mating behaviours are frequently associated with morphological differences such as body size and weapon size. Therefore, we investigated whether the behavioural phenotypes identified also exhibit morphological differences.

## Materials and methods

### Mite rearing

To initiate our culture of *T. urticae*, a sample population was obtained from a commercial producer of biological control agents (Koppert Biological Systems, The Netherlands). The mites were reared on detached kidney bean leaves, *Phaseolus vulgaris* L., on wet cotton wool in a plastic box under constant climatic conditions (25 ± 1 °C, 60 % RH and LD 16:8 h photoperiod).

### Observation of male precopulatory behaviour

To investigate differences in premating behaviour among males, we made video records of male mating behaviour of the two-spotted spider mite. We collected about 100 adult males and a single female in the last moulting stage (teleiochrysalis) from the mite culture randomly and introduced them onto a piece of kidney bean leaf (1.5 cm in diameter). We used such an unnaturally high sex ratio (normal sex ratio is around 25 % male; e.g. Young et al. 1986; Roeder 1992) to observe as much male–male interactions around the teleiochrysalis female as possible. The males’ behavioural activities near the teleiochrysalis female were recorded for 2–3 h using a recorder (JVC SR-VS 30 for MiniDV and Super VHS) and a camera (Panasonic Convertible AW-E300, Panasonic Corporation, Japan) connected to a binocular microscope (Olympus SZX12). For two behavioural phenotypes (referred to as phenotype T and S, below) we made sure to obtain at least 30 records, each lasting from spotting the male close to a teleiochrysalis until the female moulted and was mated (12 records by continuous video observation and 18 or more by time-lapse video observation). For the third behavioural phenotype (referred to as phenotype O below) 10 (continuous) records were obtained each lasting until they found a freshly emerged female to mate with or for a maximum of 2 h.

### Identification of male behavioural phenotypes

Based on the video records, we observed that some guarding males were easily disturbed by intruder males, and engaged in fights with them, but other guarding males are not (see section “Results”). This difference enabled us to establish a method to discriminate these two types of males, as follows. From the mite culture we randomly collected 20 adult males and a teleiochrysalis female with silver-like appearance indicating that it will moult in a few hours. Using a fine brush the males and female were introduced onto a leaf arena, consisting of a kidney bean leaf disc 1.5 cm in diameter placed on wet cotton wool in a petri dish. Half an hour after their introduction, observation of male behaviour started. When a male was found mounting the teleiochrysalis female, another male was gently picked up from the mite culture using a fine brush made wet to fix the mite. The brush had the male on top with its first legs freely moving and it was moved towards the mounting male close enough, to allow the first legs to touch the mounting male’s body several times. The mounting male’s response to this artificial disturbance was observed and classified in one of the two following categories. If the mounting male responded actively toward the male on the brush and showed a fighting posture (extended stylets and first pair of legs lifted up; Potter et al. 1976a, b) either before or after it left its position on the female’s dorsum, it was classified as phenotype T. If, however, it did not show a response to the artificial disturbance and stayed put, it was classified as phenotype S. After having classified the male in this way, this classification was tested by observing the behaviour of the mounting male until the teleiochrysalis female started to moult. These observations were recorded on videofilm using a camera (Leica IC80 HD, Leica Microsystems, Germany) connected to a binocular microscope and a computer with adequate software (LAS EZ software version 2.0.0, Leica Microsystems). These video records were checked for responses of the mounting male to the first three natural disturbances by males that spontaneously intruded the area bordering the teleiochrysalis (approximately with a diameter equal to the size of the teleiochrysalis female, cf. Cone 1985, Fig. 1.4.3.1). Males lacking a response to these natural disturbances were classified as phenotype S and males showing responses at least once were classified as phenotype T. This enabled us to compare the initial classification based on artificial disturbances to that based on natural disturbances. We did not carry out such classification tests for phenotype O because it is hard to define a time period without guarding behaviour that is short enough to be practical for quick classification and long enough to be a good predictor of continued absence of guarding behaviour.

### Morphology of males of phenotype T and phenotype S

The two-spotted spider mite has haplodiploid sex determination: females and males develop from fertilized eggs (2n) and unfertilized eggs (n) respectively, so that non-inseminated females produce unfertilized eggs. To prepare sufficient numbers of males, we used virgin females in this experiment. Since a difference in mating behaviour has been observed between males from virgin females and males from fertilized females (Ohzora and Yano 2008), we confirmed if the males from virgin females show the premating behaviours that we observed in the videos made earlier. We collected 20 teleiochrysalis females from our mite culture, and introduced them onto a 5 × 5 cm kidney bean leaf placed on wet cotton wool in a plastic box. They were allowed to moult to adult females and to lay unfertilized eggs for a week, and after removing the females from the leaf the unfertilized eggs were allowed to develop into adult males. We randomly collected five adult males and put them onto a bean leaf arena with a single teleiochrysalis female. Two hours after introduction of the males, the mounting male was classified by disturbing the male as described above.

The males classified in this way were put into fixation liquid (methanol:acetic acid:distilled water = 2:2:1; Saito and Osakabe 1992), because this liquid adequately fixes external tissues of mites and also prevents their legs to bend. Two to four weeks after fixation, we picked up the males from the liquid and each male was placed in a droplet of Hoyer’s solution on a slide glass and subsequently a cover glass was placed on top of the droplet. The slides were heated using a soldering iron which had the effect that the male’s legs stretched. Subsequently, a 2 ml microtube filled with water so as to obtain a total of 3 g weight was put on top of the cover glass, and the slides were kept at ca. 40 °C for 2 weeks. This weight created an even pressure on slide-mounted specimens. Using a phase contrast microscope and a microscopy digital USB camera (Optika, Italy), we took photos of the specimens first and then of an object micrometer (a transparent glass plate displaying a 1 mm long ruler with 0.01 mm scale).

**Fig. 1:**
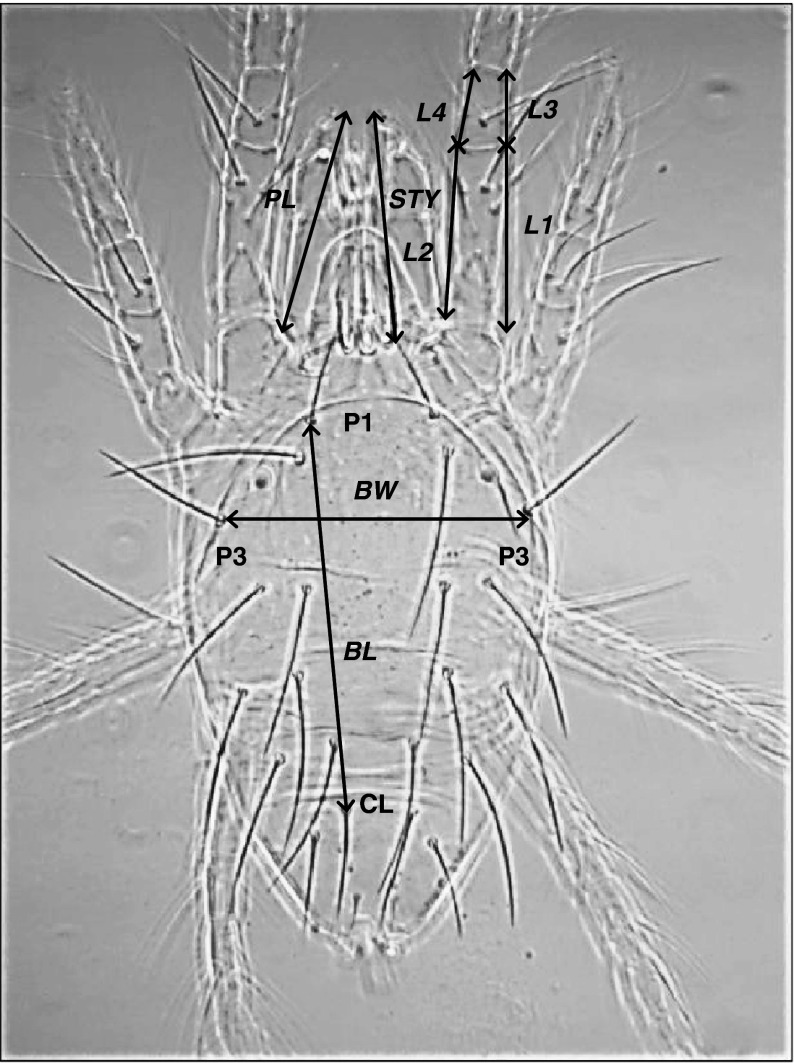
Morphological characteristics measured for *Tetranychus urticae* males. The body length (*BL*) was estimated by taking the mean of right and left distances between the bases of dorsal hairs *P1* and *CL*, the body width (*BW*) by taking the distance between *P3* dorsal hairs, the length of the first leg by taking the mean of right and left lengths of genu and femur (=(sum of *L1* − *L4*)/2), the length of the stylets (*STY*) by taking the mean value of the linear distances between the basis and the tip of the right and left stylets lengths and the length of the pedipalps (*PL*) by taking the mean of the linear distances between the basis and the tip of the right and left pedipalps

Because males are known to use their first legs, their stylets and their pedipalps during male–male combat (Potter et al. 1976a, b; Cone 1985), we measured the lengths of these structures in addition to the body width and the body length of the males (Fig. 1). For these measurements, we used a photomicrograph and image-processing software (Image J ver. 1.41; National Institutes of Health, USA). For each male mating type we established these measurements for 20 specimens.

### Statistical analysis

We tested the correspondence between male mating behaviour classified by artificial disturbance and that classified by natural disturbance, using McNemar’s Chi squared test with continuity correction, which is appropriate for count data from repeated tests on the same subjects (Sokal and Rohlf 1995).

To test morphological differences between phenotype T and phenotype S, we constructed a generalized linear model with binomial error distribution, in which the response variable was mating type (territorial or sneaker) and the explanatory variables were the measures of morphological traits of the males. The effect of each explanatory variable was tested by a likelihood ratio test to compare the model with the explanatory variable to the model without. Because there is a possibility that these explanatory variables are highly correlated, we determined correlations between body length and other measurements (body width and lengths of the first legs, stylets and pedipalps) using Pearson’s product-moment correlation, and we checked multi-collinearity in the model by calculating the values of variance inflation factors (VIF). These analyses were performed with the freeware statistical package R version 2.14.1 (R Development Team 2011).

## Results

### Male mating phenotypes

Based on the video records we described three male phenotypes of precopulatory behaviour. First, there were males exhibiting phenotype T, as shown in the video provided in supplementary materials 1 and 2. Here, males initiate mounting the dorsum of a female teleiochrysalis well before emergence of the adult female. In absence of competitors, they continue to stay on the dorsum. Sometimes, the males stop staying on the female dorsum and weave a web of silk from the dorsum of the teleiochrysalis to the substrate, but thereafter they return to stay on the female dorsum again. In presence of competitors, however, the males are easily disturbed by approaching adult males (intruders), engage themselves in fights with them and, after driving them off, they tend to return to the teleiochrysalis female.

Second, we observed males exhibiting phenotype S, as shown in supplementary materials 3 and 4. Just like phenotype T, these males initiate mounting behaviour well before emergence of the adult female. However, these males differ from phenotype T in that they are not disturbed by intruders: they do not engage in fights with them nor are they attacked by the intruder.

Third, we observed 10 males that wandered around and did not show mounting and guarding behaviour. Most of these males (8) continued to move until they found a mating opportunity with a female that just moulted (phenotype O), whereas the remainder (2) continued until we stopped the observation after 2 h.

### Discrimination between behavioural phenotypes of males

Most of the males that had been classified as phenotype T (since they responded to artificial disturbance), also showed responses to natural disturbance. Only in one case we identified a male as phenotype T based on artificial disturbance, whereas this male did not respond to three consecutive natural disturbances by rival males. In this particular case, however, the rival males may have been phenotype S males because they did not show any responses to disturbance by other males. All males, that had been classified as phenotype S according to the artificial disturbance test, did not show any response to the first three intruder males (representing natural disturbance). Hence, overall, there was only one mismatch in classifying male phenotype according to artificial and natural disturbance (Table 1), and there was no significant difference in the classification between these two methods (McNemar’s Chi squared test: *χ* ^2^ = 0, *df* = 1, *P* = 1). We conclude that identifying phenotypes of male mating behaviour by using artificial disturbance tests is a reliable method.

**Table 1:** Relationship between classifications of male phenotype in mating behaviour in response to artificial disturbance and to natural disturbance

	*Classification artificial disturbance*	
	Phenotype T	Phenotype S
*Classification natural disturbance*		
Phenotype T	20	0
Phenotype S	1	22

As explained in the M&M section, we did not carry out tests to quickly identify phenotype O because it is practically not feasible. So, the existence of this behavioural phenotype is only based on the observation that some males continue to search for a long time without guarding a teleiochrysalis female.

### Morphology of male of phenotype T and phenotype S

**Fig. 2:**
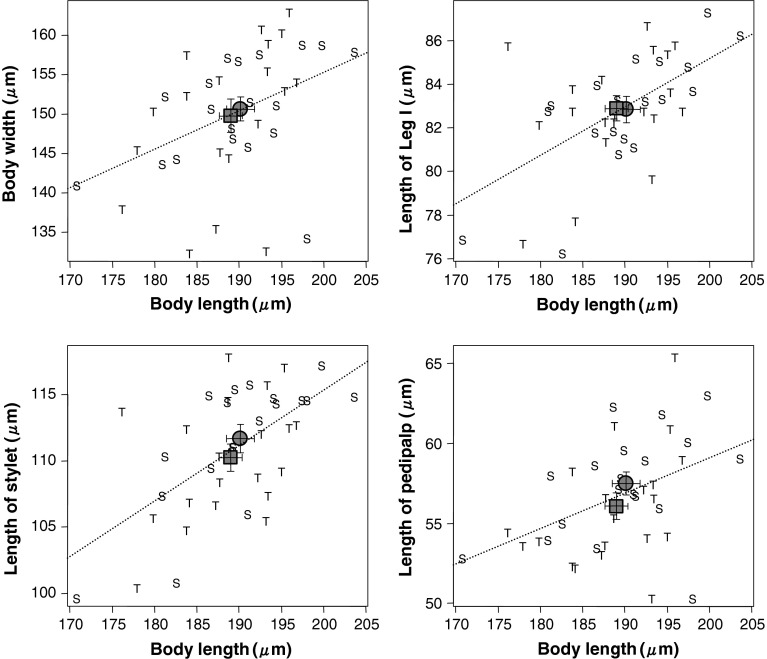
Relations between body length and other measured make characteristics (body width and lengths of leg I, stylet and pedipalp). “T” and “S” represent males of phenotype T and males of phenotype S, respectively (n = 20 in each). *Filled square* and *filled circle* indicate the mean for the males of phenotype T and males of phenotype S, respectively. *Error bars* indicate standard errors of the means. The *dotted lines* represent regression lines between body length and other characteristics of male morphology

There were significant correlations between body length and other measurements (body width: *r* = 0.415, *df* = 38, *P* < 0.01; length of leg I: *r* = 0.577, *df* = 38, *P* < 0.001; length of stylet: *r* = 0.578, *df* = 38, *P* < 0.001; length of pedipalp: *r* = 0.431, *df* = 38, *P* < 0.01; Fig. 2). However, values of VIF did not exceed 10 (body length: 1.737, body width: 2.331, length of leg I: 2.997, length of stylet: 3.417, length of pedipalp: 2.597). Hence, we constructed a generalized linear model with binomial error distribution to compare male morphology. This yielded no significant difference in any of the morphological variables between males of phenotype T and phenotype S (Table 2; Fig. 2). Thus, we have no reason to assume that the behavioural phenotypes correspond to different morphologies.

**Table 2:** Results of likelihood ratio tests using a generalized linear model with binomial error distribution, in which the response variable was male mating phenotype (T or S) and the explanatory variables were measures of morphological traits of the males

Variables	*df*	LRT	*P*
Body length	1	0.020	0.89
Body width	1	0.037	0.85
Length of leg I	1	0.655	0.42
Length of stylet	1	0.479	0.49
Length of pedipalp	1	0.524	0.47

LRT indicates log-likelihood ratio statistic

## Discussion

Whereas fighting with rival males as well as teleiochrysalis mounting and guarding have been described extensively for males of the two-spotted spider mite (Potter et al. 1976a, b), they were recorded as behavioural events preceding actual copulation. Here, we show that male spider mites consistently show one of three types of precopulatory behaviour for at least a few hours. These behavioural syndromes shown to be repeatable over a short-term are referred to here as behavioural phenotypes. Many male spider mites exhibit phenotype T: they first search for a moulting female and, upon finding one, they mount her dorsum, but leave that position on the dorsum to fight off rival males and return to that position when rivals do not show up again. We interpret phenotype T as that of males exhibiting territorial behaviour. In contrast to phenotype T, we provide first evidence for phenotype S whereby spider-mite males also mount teleiochrysalis females but stay put despite multiple disturbances even in the presence of potential rival males. This behavioural phenotype stands out as unique not only in that it lacks a response to the rival males, but also in that it is not approached aggressively by rival males. Moreover, males with this newly discovered phenotype S do become active at the moment when the adult female emerges from the teleiochrysalis and they do gain reproductive success depending on their proximity to the moulted female (Sato et al., unpublished data). Therefore, we characterize this behavioural phenotype as being stealthy and refer to it as sneaking behaviour, as this term has been used to describe similar phenomena in many other animals (e.g. side-blotched lizard; Sinervo and Lively 1996, salmon and trout; Esteve 2005, three-spined stickleback; van den Assem 1967, horseshoe crab; Brockmann et al. 1994, sunfish; Gross 1982).

Our results raise the question: how can sneaker males escape from competition with rival males? As a first hypothesis, suppose spider mite males cannot detect the sex and the stage of another individual spider mite at contact and can only explore it by tactile means. Then, it can only judge the quality of the individual contacted from the aggressiveness of its responses. If there is a vigorous counterattack, it will consider it to be a rival male, but if not it can be anything else, e.g. a female or a juvenile. Sneaker males may take advantage of this situation because by not responding to contact, they signal not to be a potential competitor for mates. As a second hypothesis, suppose spider mite males do not see (they have primitive eyes but cannot form images; McEnroe and Dronka 1969), but they can smell. This is a realistic assumption because males have olfactory sensors and they have been shown to respond to sex pheromones released by females (Royalty et al. 1992; Margolies and Collins 1994). In that case, sneaker males can only go unnoticed by rival males if they can mimic the odour of a female (e.g. pheromone). Future experiments are needed to test these two hypotheses on sneaker mechanisms against each other.

In addition to phenotype T expressing territorial behaviour and phenotype S expressing sneaking behaviour, we also suspect a third phenotype O, expressing opportunistic behaviour. It should be emphasized that our description of the opportunistic mating phenotype is largely conjectural. We do not have evidence for male spider mites with a phenotype that gives priority to searching for direct mating opportunities instead of guarding potential mates. In fact, to provide evidence for such behaviour it is necessary to follow wandering males on a leaf for periods of time much longer than the maximally 2–3 h we observed them. This is not easily achieved even with the use of time-lapse video techniques, because the males wander around and this can only be observed at a relatively lower magnification level. However, it may be possible to score the frequency of non-mounting in the presence of teleiochrysalis females that are not guarded by a male.

In most cases, alternative types of male mating behaviour are associated with morphological differences such as body size and weapon size. For example, dung beetle males show a dimorphism in the pair of horns that protrude from the base of their head (Emlen 1997). Body size and relative horn length affect the outcome of male–male combat, and large, horned males fight with other males for females, but small, hornless males exhibit sneaking behaviour (Emlen 1997). Morphological differences in relation to mating behaviour are also observed in several species of mites. In the bulb mite, *Rhizoglyphus robini,* there are two types of males, ‘fighters’ and ‘scramblers (non-fighters)’: a ‘fighter’ has a thickened and sharply terminated third pair of legs and can kill other males, but ‘scramblers’ have unmodified legs and they are defenseless (Radwan 1995, 2003; Smallegange 2011a, b; Smallegange and Coulson 2011). Similar male dimorphisms were observed in the acarid mite, *Caloglyphus berlesei* (Radwan 1993). However, we could not find any morphological differences between territorial and sneaker males in the two-spotted spider mite (Table 2; Fig. 2). Larger males usually win male–male combat in this species (Potter et al. 1976a, b; Enders 1993). Since fighting males use their first legs, stylets and pedipalps, it is likely that the size of these structures affects the outcome of male–male combat. We therefore hypothesized that sneaker males are smaller than territorial males. However, we found no morphological differences between territorial and sneaker males and suggest that there are other factors determining who wins the combat.

Although polymorphism in male morphology helps detecting alternative male mating behaviours, there are studies reporting alternative male mating behaviours without morphological differences. For example, in the horseshoe crab, *Limulus polyphemus,* males change their mating behaviour depending on their age (Brockmann et al. 1994). Males usually couple with a female by grasping the female’s terminal spines, and these male–female pairs travel together to the nesting beach. However, older males in relatively poor condition (because they have deteriorated or are parasitised) exhibit satellite behaviour: they gather around nesting couples, and release sperm that fertilize part of the eggs released by the female in the couple. In future experiments, our aim will be to test whether alternative phenotypes of male mating behaviour in the two-spotted spider mite are genetically fixed and possibly represent personalities (repeatable behavioural differences between individuals; Sih et al. 2004; Réale et al. 2007; Dall et al. 2004) or whether they are plastic in that they depend on rival male density or on age (as in the horseshoe crab) (Sato et al., in prep.).

## Electronic supplementary material

Below is the link to the electronic supplementary material.

Supplementary material 1 (MPG 8516 kb)

Supplementary material 2 (MPG 3898 kb)

Supplementary material 3 (MPG 11724 kb)

Supplementary material 4 (MPG 14872 kb)
